# Temperature-Dependent Active-Site Rearrangements of PETaseSM14: Insights from Molecular Dynamics Simulations

**DOI:** 10.3390/ijms27062825

**Published:** 2026-03-20

**Authors:** Ki Hyun Nam

**Affiliations:** College of General Education, Kookmin University, Seoul 02707, Republic of Korea; structure@kookmin.ac.kr

**Keywords:** polyethylene terephthalate, PETase, substrate binding cleft, temperature, molecular dynamics simulation

## Abstract

Polyethylene terephthalate (PET) is a synthetic polymer that is widely used in the production of textiles, packaging materials, and beverage bottles. However, its high durability and resistance to abiotic degradation result in serious environmental and health problems. PETase is an enzyme that can depolymerize PET into value-added products, thereby providing an environmentally friendly strategy for PET recycling. PETaseSM14 from a marine sponge, *Streptomyces* sp. SM14, has a high salt tolerance and thermal stability, thus suggesting its potential for PET degradation applications. However, the substrate recognition mechanism of PETase remains unclear because the catalytic residue is buried within residues that form the substrate-binding cleft. To elucidate the molecular mechanism of PETaseSM14, all-atom molecular dynamics simulations were performed at 300, 320, and 340 K. The results revealed that the overall α/β fold remained stable at all temperatures, whereas temperature-dependent local fluctuations and conformational changes were observed in the substrate-binding cleft and N-terminal region. At 300 and 320 K, positional shifts and conformational changes in Tyr88 exposed the catalytic Ser156 to the solvent, thereby forming a potential substrate-binding cleft. In contrast, at 340 K, which is higher than the melting temperature of PETaseSM14, disruption of the charge-relay system of the catalytic triad occurs through conformational changes in His234. Substantial temperature-dependent conformational and positional changes in the N-terminal region of PETaseSM14 were observed at 320 and 340 K. These results provide mechanistic insight into the temperature-dependent active-site rearrangements and offer rational engineering strategies to enhance the efficiency of PETase for PET biodegradation.

## 1. Introduction

Polyethylene terephthalate (PET) is one of the most widely produced synthetic polymers worldwide, and it is extensively used in textiles, packaging, and beverage bottles because of its resistance to corrosion, low density, cost-effectiveness, and excellent chemical and physical stability [1,2,3]. However, its high durability and resistance to abiotic decomposition result in the continuous environmental accumulation thereof [1,2,3]. Traditional recycling methods, such as mechanical reprocessing and thermochemical treatment, are limited by the degraded material properties and high energy demands, thus motivating the development of biological PET recycling approaches that can depolymerize PET into its monomeric constituents under mild conditions [3,4,5].

**Figure 1:**
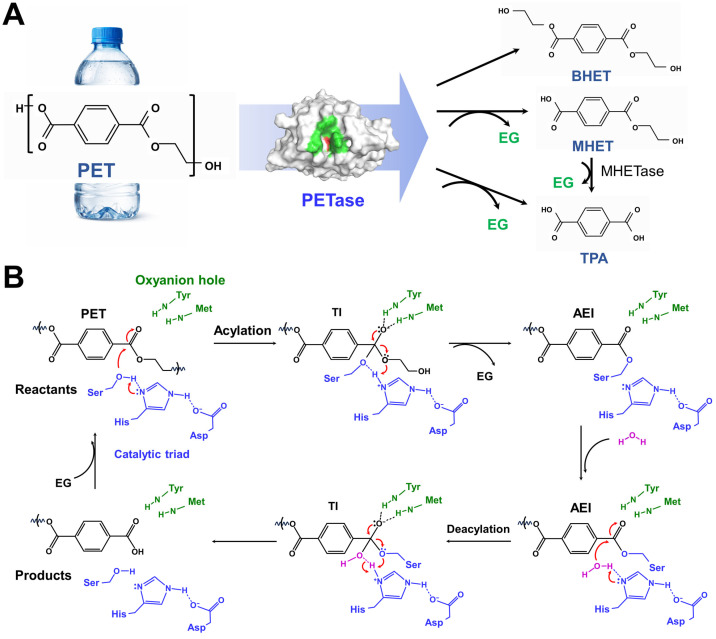
Schematic diagram of PET degradation and the catalytic mechanism of PETase. (**A**) Depolymerization of PET by PETase produces BHET, MHET, and TPA. The PET bottle image was generated using ChatGPT-5.2. (**B**) Reaction mechanism of PET degradation catalyzed by PETase. TI, tetrahedral intermediate; AEI, acyl-enzyme intermediate. Red arrows indicate the electron transfer pathway. The reaction mechanism and figure style were adapted from previous studies [12,13] and modified here.

A pivotal breakthrough in this field was the discovery of *Ideonella sakaiensis* 201-F6, a bacterium capable of using PET as an energy and carbon source by secreting PETase, a PET-hydrolyzing enzyme [6]. This enzyme catalyzes the hydrolytic cleavage of ester bonds in PET and produces mono-(2-hydroxyethyl) terephthalate (MHET), terephthalic acid (TPA), and bis(2-hydroxyethyl) terephthalate (BHET) [6,7,8] (Figure 1A). Structural and mechanistic studies have revealed that PETase belongs to the α/β-hydrolase family and exhibits high substrate specificity and functions via a two-step acylation and deacylation mechanism mediated by a Ser–His–Asp catalytic triad, with substrate binding facilitated by an active-site architecture that is adapted to interact with the aromatic and ester moieties of PET [9,10,11,12] (Figure 1B).

PETase exhibits higher specificity and catalytic activity toward PET than other previously identified hydrolases, such as cutinases and lipases, which suggests its potential use for environmentally compatible bioprocessing [7,14]. However, wild-type PETase exhibits limited efficiency toward highly crystalline commercial PET substrates, thus prompting extensive research into protein engineering, including site-directed mutagenesis as well as computational design, to enhance its catalytic performance, thermostability, and substrate affinity for practical recycling applications [9,15,16,17,18]. Accordingly, various PETase variants with substantially improved depolymerization activity and thermostability have been developed, suggesting that these engineered biocatalysts could play a transformative role in sustainable enzyme-based PET recycling technologies [9,15,16,17,18]. Therefore, understanding the structural mechanism of PETase is important for developing more efficient enzymes for practical applications [19].

A PETase-like enzyme, designated PETaseSM14, was discovered from the marine sponge-derived *Streptomyces* sp. SM14 [20,21]. This enzyme efficiently catalyzes post-consumer plastic substrates and exhibits a high salt tolerance (up to 1.5 M) and heat resistance (T_m_ = 56.26 °C) [22], which suggests that it has potential for PET waste bioremediation. The crystal structure of PETaseSM14 revealed a typical α/β-hydrolase fold containing a catalytic triad (Ser156–Asp202–His234), which mediates nucleophilic attack and proton relay during catalysis [22]. Based on structural comparisons, aromatic and hydrophobic residues, including Tyr88, His155, Met157, Trp181, and Ile204, were identified within the substrate-binding cleft, where they stabilize the PET aromatic moieties [22]. Interestingly, the position and overall conformation of the catalytically active site in PETaseSM14 were highly conserved compared to that of IsPETase; however, the residues constituting the substrate binding cleft exhibited different positions and conformations [22]. The substrate-binding cleft of PETaseSM14 revealed a more closed state than that of IsPETase, which may limit the substrate accessibility. Recently, a molecular dynamics (MD) simulation study demonstrated that the width of the substrate-binding cleft between Tyr88 and Trp181 of PETaseSM14 increased slightly by approximately 2 Å under 900 mM NaCl conditions [23]. This result indicates that the substrate-binding region can be affected by environmental conditions. Nevertheless, when considering the MHET-bound state of IsPETase, which exhibits an open conformation of the substrate-binding cleft for MHET binding [24], conformational changes in the substrate-binding site of PETaseSM14 may be essential for substrate recognition of PET; however, the underlying mechanism has not yet been fully elucidated.

To better understand the molecular function of PETaseSM14 and provide insights for future protein engineering, MD simulations of PETaseSM14 were performed at 300, 320, and 340 K. The molecular flexibility of the MD simulation trajectories was then analyzed and compared. Temperature-dependent rearrangements of the substrate-binding cleft were investigated, with particular focus on the flexible N-terminal region. This study provides an understanding of the temperature-dependent structural rearrangements in the substrate-binding cleft of PETaseSM14 and offers valuable information for future protein engineering of PETase.

## 2. Results

### 2.1. Revisiting the Structures of PETaseSM14 and IsPETase

**Figure 2:**
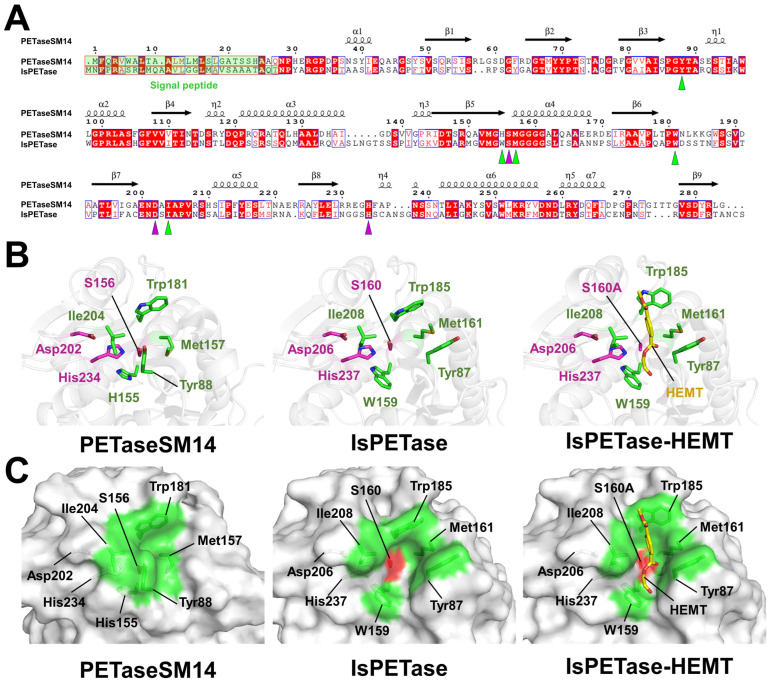
Amino acid and structural comparison of the substrate binding cleft of PETaseSM14 (PDB code: 9HYD) with that of native IsPETase (5XJH) and HEMT-bound IsPETase (5XH3). (**A**) Structure-based sequence alignment of PETaseSM14 (UniProt: A0A679PDB4) with IsPETase (A0A0K8P6T7). The residues involved in the catalytic triad and substrate binding cleft are indicated by magenta and green triangles, respectively. (**B**) Cartoon and (**C**) surface representations of PETaseSM14, native IsPETase, and HEMT-bound IsPETase. The residues involved in the catalytic residue and substrate binding cleft are indicated in red and green, respectively.

To understand the molecular mechanism of PETaseSM14, the previously reported crystal structure of PETaseSM14 (PDB code: 9HYD) [22] was compared with those of the previously determined native IsPETase (5XJH) [10] and 1-(2-hydroxyethyl)-4-methyl terephthalate (HEMT)-bound IsPETase (5XH3) [24]. Sequence alignment between PETaseSM16 and IsPETase revealed a moderate sequence identity of approximately 43% over 263 aligned residues, excluding the signal peptide. The catalytic residues were strictly conserved, and most of the substrate-binding residues were also conserved (Figure 2A). The superposition of PETaseSM14 with the native and HEMT-bound IsPETase structures revealed high structural similarity, with RMSDs of 0.658 Å and 0.694 Å, respectively (Supplementary Figure S1). The main-chain positions of the residues involved in the catalytic triad and substrate-binding cleft of PETaseSM14 and IsPETase were almost identical. The distance between the OD2 atom of Asp202 and the ND1 atom of His234 in the catalytic triad of PETaseSM14 was 2.23 Å, whereas the corresponding distances in native IsPETase and HEMT-bound IsPETase were 2.67 Å and 2.69 Å, respectively. Similarly, the distance between the side chains of the NE2 atom of His234 and the OG atom of Ser156 in the catalytic triad of PETaseSM14 was 2.68 Å, which was comparable to the corresponding distance of 2.92 Å in IsPETase; however, the active Ser156 in HEMT-bound IsPETase was substituted with alanine. This indicated that the catalytic triad residues in PETaseSM14 and IsPETase exhibited identical configurational interactions within the catalytic triad with a stable hydrogen-bond network, thus suggesting that the catalytic triad is highly structurally conserved. In contrast, the side-chain residue conformations involved in the substrate-binding cleft differed significantly between PETaseSM14 and IsPETase (Figure 2B). In PETaseSM14, the Tyr88 phenolic ring was oriented toward the catalytic Ser156 residue. The closest distance between the OG atom of Ser156 and the CD1 atom of the phenolic ring of Tyr88 was 3.41 Å (Figure 2B). In addition, the distance between the CG atom of Tyr88 and the CB atom of Ile204 of PETaseSM14 was 5.74 Å. The side chains of Tyr88, Met157, Trp181, Ile204, and His234 in PETaseSM14 were positioned close to the catalytic residue Ser156. Meanwhile, the tyrosine side chain of Tyr87 in IsPETase was oriented away from the active site, and the closest distance between the OG atom of Ser160 and the CG atom of the phenolic ring of Tyr87 was 7.68 Å (Figure 2A). The distances between the CG atom of Tyr87 and the CB atom of Ile208 in the native and HEMT-bound IsPETase were 9.17 and 9.51 Å, respectively, which resulted in the exposure of the catalytic Ser160 residue to the surface.

Surface representation revealed that the active site of PETaseSM14 was buried by residues that form the substrate-binding cleft, particularly Tyr88, thus indicating that the active site may not be readily accessible to PET substrates (Figure 2C). In contrast, the substrate-binding cleft of IsPETase is clearly formed, which allows substrates such as PET to bind within the open cleft, accompanied by conformational changes in the Trp185 residue (Figure 2C). These analyses indicated that residues forming the substrate-binding cleft of PETaseSM14 require considerable rearrangement to create a substrate-binding cleft that is comparable to that of IsPETase.

### 2.2. Temperature-Dependent Structural Change in PETaseSM14

Temperature is a critical factor that modulates the enzyme structure and catalytic function [25,26,27]. To investigate the effect of high temperatures on the PETaseSM14 structure, all-atom MD simulations were performed at 300, 320, and 340 K, which correspond to the low-activity, near-optimal, and higher than the melting point temperatures (Tm), respectively [22]. During the MD simulation, the systems were neutralized with counter ions to investigate only the temperature effect on PETaseSM14, and additional variables such as high salt concentration were not applied.

**Figure 3:**
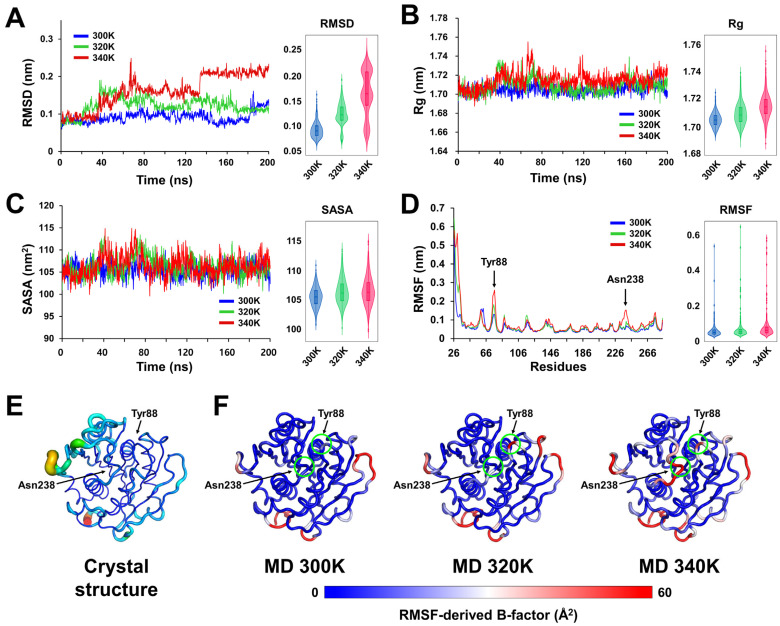
Temperature-dependent molecular dynamics of PETaseSM14. (**A**) RMSD, (**B**) Rg, (**C**) SASA, and (**D**) RMSF obtained from MD simulations at 300 K (blue), 320 K (green), and 340 K (red). (**E**) B-factor putty representation of the crystal structure of PETaseSM14 (PDB code: 5XJH). (**F**) RMSF-derived B-factors of PETaseSM14 from MD trajectories at 300, 320, and 340 K.

RMSD analysis revealed that at 300 K, PETaseSM14 exhibited a stable RMSD of approximately 0.8–1.0 nm for most of the simulation (Figure 3A). At around 180 ns, the RMSD increased to approximately 1.5 nm; however, the overall low RMSD values and small fluctuations indicate that the global fold of the PETaseSM14 enzyme remained stable without major deviation from the initial structure. At 320 K, the RMSD initially increased from approximately 1.0 to 1.8 nm during the first 0–40 ns (Figure 3A). After this initial rise, the RMSD gradually decreased and ultimately reached a fluctuation range similar to that observed at 300 K, thus indicating that the structure stabilized after an initial conformational adjustment. At 340 K, the RMSD gradually increased until approximately 60 ns and stabilized at around 1.5 nm (Figure 3A). However, after approximately 130 ns, the RMSD further increased to approximately 2 nm and then remained stable at this elevated level, which suggests the occurrence of a significant conformational transition. Overall, these RMSD analyses demonstrate that PETaseSM14 maintains a stable global structure at 300 and 320 K, with only modest thermal fluctuations, whereas at 340 K, the protein undergoes substantial conformational changes.

Rg analysis demonstrated that PETaseSM14 at 300 K remained at approximately 1.69–1.71 nm for most of the simulation, with only small fluctuations, thus indicating that it has a compact overall structure (Figure 3B). Meanwhile, the Rg values of PETaseSM14 at 320 and 340 K increased slightly and fluctuated mainly within approximately 1.69–1.72 and 1.70–1.73 nm, respectively (Figure 3B), thereby indicating enhanced conformational breathing but without a significant protein expansion. Similarly, the SASA of PETaseSM14 showed a slight increase in fluctuations as the temperature increased from 300 to 340 K (Figure 2C); however, the overall SASA values (approximately 103–108 nm^2^) remained comparable across all temperatures, which indicates that the solvent-exposed surface area of the protein did not undergo large-scale changes.

RMSF analysis revealed that the fluctuations of the N-terminal region of PETaseSM14 increased significantly with increasing temperatures (Figure 3D). In contrast, the core region exhibited similar RMSF values at 300 K, 320 K, and 340 K, except for Tyr88, which is involved in substrate recognition, and Asn238, which is located near the substrate-binding cleft. For Tyr88, the RMSF progressively increased with the temperatures, whereas Asn238 displayed a significant increase in fluctuation specifically at 340 K. The B-factor putty representation of the crystal structure of PETaseSM14 (PDB code: 9HYD) demonstrated that Tyr88 and Asn238 adopt relatively rigid conformations in the static structure (Figure 3E). In contrast, the RMSF-derived B-factors from MD simulations at 300 K, 320 K, and 340 K revealed temperature-dependent increases in the flexibility of the Tyr88 and Asn238 regions (Figure 3F). Taken together, these MD analyses showed that PETaseSM14 exhibits a thermally robust global fold across a wide temperature range, while the local, functionally relevant regions become increasingly flexible at elevated temperatures. 

### 2.3. Temperature-Dependent Conformational Changes in the Active Site and Substrate-Binding Cleft

**Figure 4:**
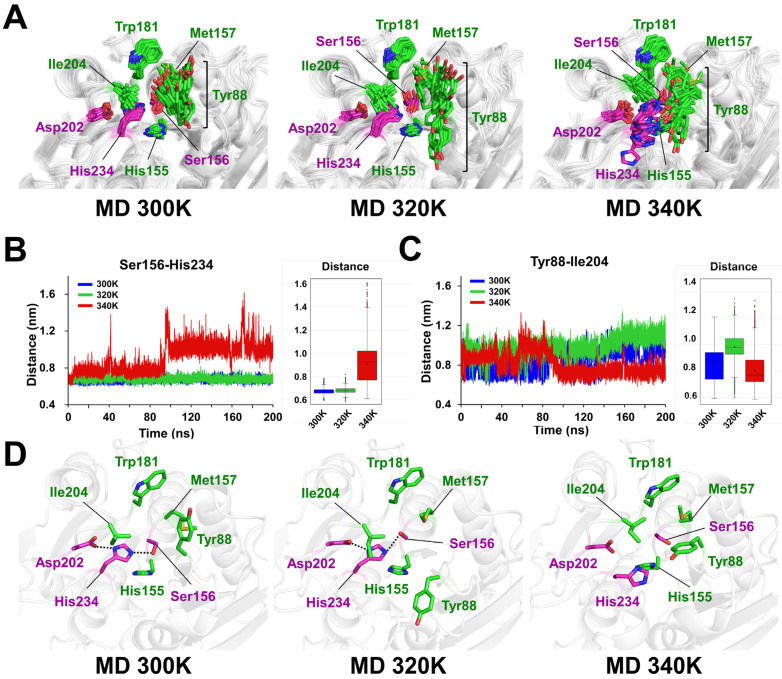
Analysis of the MD trajectories of PETaseSM14 at 300, 320, and 340 K. (**A**) Temperature-dependent dynamic ensemble of the substrate-binding pocket of PETaseSM14 at 300, 320, and 340 K. Analysis of the distance time series of (**B**) Ser156 and His234 forming the catalytic triad and (**C**) Tyr88 and Ile204 defining the substrate-binding cleft. (**D**) Cartoon representations of the representative MD structure of PETaseSM14 at 300, 320, and 340 K. The catalytic triad residues are highlighted in magenta.

To understand the temperature-dependent conformational change in the active site and substrate binding cleft, structures were extracted at regular intervals from the MD trajectories at 300 K, 320 K, and 340 K and then superimposed. At 300 K, the catalytic triad exhibited a stable hydrogen-bond network (Figure 4A). Moreover, Tyr88 exhibited a significant positional shift, and subtle movements of the substrate-binding residues Trp181 and His234 were observed, along with minor side-chain conformational changes in Tyr88, Met157, and Ile204 (Figure 4A). At 320 K, the catalytic triad maintained a stable hydrogen-bond network (Figure 4A). A significant positional shift in the main chain and a conformational change in the Tyr88 phenyl ring were observed, in which Tyr88 moved away from the active site, thereby exposing the catalytic Ser156 to the surface (Figure 4A). In addition, conformational changes in the side chains of Met157 and Ile204 were observed, although these changes were smaller than those of Tyr88, and Trp181 and His234 only exhibited subtle positional shifts (Figure 4A). At 340 K, His234, which is involved in the catalytic triad, underwent a significant conformational change, which disrupted the hydrogen-bond network of the Asp–His–Ser catalytic triad (Figure 4A). This rearrangement of His234 affected the local environment of the neighboring residues, including Tyr88, Ile204, and Asn238, indicating disruption of the active-site architecture at this temperature. Despite these local changes, the overall fold of the protein remained largely preserved, except for the N-terminal region.

The analysis of the distance time series between Ser156 and His234, which are involved in the catalytic triad, revealed that the charge-relay system was stably maintained at 300 and 320 K (Figure 4B). In contrast, at 340 K, the distance between Ser156 and His234 was relatively larger than that at the other temperatures, with a significant increase in the distance observed after 90 ns of simulation (Figure 4B). The center-of-mass (COM) distances between Ser156 and His234 in the MD trajectories of PETaseSM14 at 300, 320, and 340 K were 6.72 ± 0.22, 6.80 ± 0.23, and 9.09 ± 1.51 Å, respectively (Figure 4B). Furthermore, analysis of the distance time series between Tyr88 and Ile204, which determines the width of the substrate-binding cleft, demonstrated that the substrate-binding cleft increased at 300 and 320 K, whereas at 340 K, the cleft initially increased but subsequently returned to a distance level similar to that of the initial state (Figure 4C). The COM distances between Tyr88 and Ile204 in the MD trajectories of PETaseSM14 at 300, 320, and 340 K were 8.49 ± 1.13, 9.83 ± 0.98, and 8.05 ± 1.10 Å, respectively, thus indicating that the substrate-binding cleft was wider at 300 and 320 K (Figure 4C).

Next, representative structures of the most dominant clusters from the MD simulations at 300, 320, and 340 K were analyzed (Figure 4D). In the MD structures at 300 and 320 K, the catalytic triad formed a charge-relay hydrogen-bond network. In contrast, in the MD structure at 340 K, the main chain shifted toward the solvent region by 1.98 Å, and the side chain of His234 was rotated by approximately 60° toward the solvent, thereby disrupting the catalytic triad’s hydrogen-bond network (Figure 4D). In the MD structure of PETaseSM14 at 300 K, the main and side chains of Tyr88 were shifted away from the active site by approximately 1.73 and 4.15 Å, respectively, compared with the crystal structure, and the distance between the CB atoms of Tyr88 and Ile204 increased to 8.31 Å, which resulted in the exposure of the catalytic Ser156 to the surface (Figure 4D). At 320 K, the main chain of Tyr88 was shifted away from the active site by 2.97 Å, and the Tyr88 phenyl ring was rotated by approximately 120°. Accordingly, the distance between the CG atom of Tyr88 and the CB atom of Ile204 increased to 9.68 Å, resulting in the exposure of the catalytic Ser156 to the surface (Figure 4D). In contrast, at 340 K, the Tyr88 phenyl ring was positioned above the catalytic Ser156, and the distance between the CG atom of Tyr88 and the CB atom of Ile204 decreased to 7.79 Å, which resulted in the burial of the catalytic Ser156 from the solvent.

### 2.4. HEMT Docking of the Experimental and MD Structures of PETaseSM14

**Figure 5:**
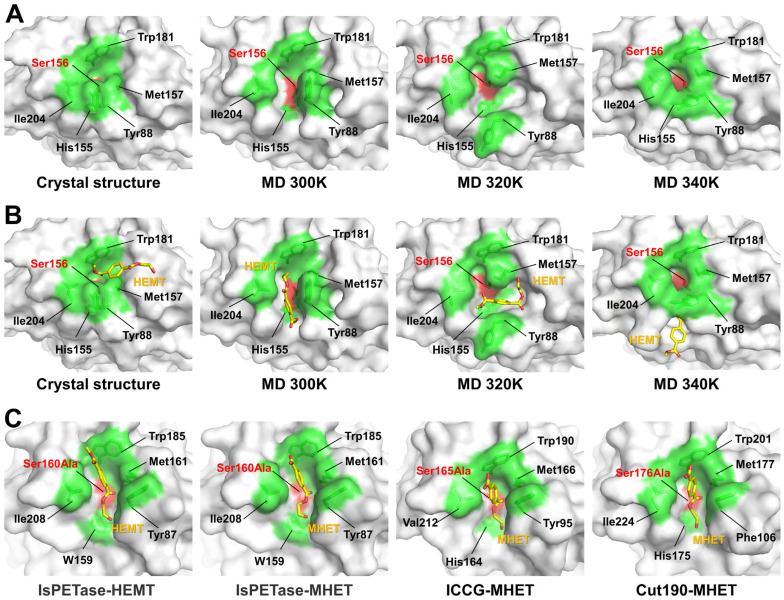
Docking of HEMT into experimental and MD structures of PETaseSM14. (**A**) Surface structures of the crystal structure of PETaseSM14 and the representative MD structures of PETaseSM14 at 300, 320, and 340 K. The catalytic residue Ser156 and substrate-binding residues are highlighted in red and green, respectively. (**B**) Structure-based blind docking of the HEMT molecule to the crystal structure and representative MD structures at 300, 320, and 340 K. (**C**) Surface structures of HEMT-bound IsPETase (PDB code: 5XH3) and MHET-bound IsPETase (7XTW), ICCG (7VVE), and Cut190 (8Z2G).

To evaluate substrate accessibility, surface structures of the experimental and MD structures of PETaseSM14 were analyzed. In the crystal structure, Ser156 is buried by neighboring residues that form the substrate-binding cleft, indicating that the PET substrate is not accessible (Figure 5A). In the surface representations of MD structure at 300 and 320 K, Ser156 was exposed on the surface, and the substrate-binding cleft was open, although its shape differed between 300 and 320 K because of different positions and orientations of Tyr88 in the representative structures (Figure 5A). Notably, the substrate-binding cleft of the representative MD structure at 300 K was similar to that of IsPETase (Figure 1 and Supplementary Figure S2). In contrast, the catalytic residue Ser156 was buried by residues forming the substrate-binding cleft in the MD structure at 340 K (Figure 4A). The calculated cavity volumes of the substrate-binding cleft in the representative MD structures of PETaseSM14 at 300, 320, and 340 K were 324, 307, and 167 Å^3^, respectively, whereas the cavity volume of the substrate-binding cleft in the crystal structure of PETaseSM14 was 80 Å^3^ (Supplementary Figure S3).

Analysis of the substrate-binding cleft showed that the MD structures of PETaseSM14 at 300 and 320 K exhibit a substrate-accessible cleft. To verify whether these conformational changes indeed generate sufficient space for substrate access, structure-based blind docking of HEMT, a PET hydrolysis intermediate, was performed using the experimental and representative MD structures of PETaseSM14 (Figure 5B and Table S1). The results showed that the HEMT molecule was well docked into the substrate-binding cleft of the MD structures of PETaseSM14 at 300 and 320 K, whereas HEMT was only partially accommodated in the substrate-binding cleft of the crystal structure of PETaseSM14 and the MD structure at 340 K due to the closed conformation of the substrate-binding cleft. These results indicate that the representative MD structures of PETaseSM14 at 300 and 320 K exhibit a PET-substrate-accessible substrate-binding cleft. In particular, the position of HEMT in the MD structure at 300 K showed rough similarity to that observed in other substrate-bound PETase-like proteins, such as HEMT-bound IsPETase (PDB code: 5XH3) and MHET-bound IsPETase (7XTW), ICCG (7VVE), and Cut190 (8Z2G) (Figure 5C). Although detailed substrate interactions and orientations between PETaseSM14 and HEMT differed from those in other experimental structures due to variations in amino acid sequence, substrate type, and side-chain conformations, these docking results nevertheless clearly suggest that temperature-induced rearrangement of the substrate-binding cleft facilitates access of the PET substrate. 

Meanwhile, the binding orientation of HEMT in the MD structure at 320 K differed from that observed in PETase–substrate complex structures. In particular, rotation of the side chain of Tyr88, which is involved in substrate recognition, altered the shape of the substrate-binding pocket at 320 K, resulting in a different binding pose compared with that at 300 K. This observation indicates that not only the positional shift in Tyr88 but also the rotation of its side chain plays an important role in accurate substrate recognition.

### 2.5. Temperature-Dependent Conformational Changes in the N-Terminal Region

**Figure 6:**
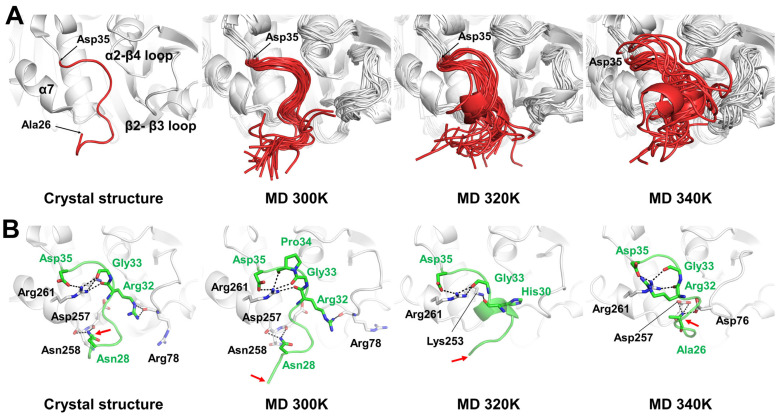
Analysis of the N-terminal region flexibility of PETaseSM14. (**A**) Temperature-dependent dynamic ensembles of the N-terminal region of PETaseSM14 from MD simulations compared with the crystal structure of PETaseSM14 (PDB code 9HYD). (**B**) Close-up views of the N-terminal region in the crystal structure and representative MD structures of PETaseSM14 at 300, 320, and 340 K. The N-terminal region is shown in green, and hydrogen-bond interactions are indicated by dotted lines.

Identification of the unfolding regions at high temperatures by MD simulation provides insight into potential protein engineering strategies for enhancing thermostability [28,29,30]. DSSP analysis showed that the MD structures of PETaseSM14 exhibited a stable overall α/β fold at all target temperatures (Supplementary Figure S4), whereas the N-terminal region exhibited significant fluctuations (Figure 3D). In the Ala26–Asp35 N-terminal region of the crystal structure of PETaseSM14 (PDB code: 9HYD), Ala26 and Gln27 exhibited high flexibility with B-factor values of 69.67 Å^2^, whereas the Asn28–Asp35 residues revealed lower flexibility with an average B-factor of 24.74 Å^2^, which was comparable to that of the whole protein at 25.16 Å^2^. In contrast, the MD simulations showed that PETaseSM14 exhibited a significant increase in N-terminal fluctuation as the temperature increased. The average RMSF values of the Ala26–Glu31 N-terminal region at 300, 320, and 340 K were approximately 0.24, 0.39, and 0.51 nm, respectively, thus indicating enhanced flexibility of the N-terminus at elevated temperatures (Figure 3D). The dynamic ensemble of the N-terminal region of PETaseSM14 was extracted at regular time intervals from the MD trajectories at 300, 320, and 340 K and exhibited temperature-induced positional and conformational changes (Figure 6A). The predominant conformation of the N-terminal region of PETaseSM14 at 300 K was similar to that of the crystal structure, whereas the N-terminal regions at 320 and 340 K differed substantially (Figure 6A). To investigate the temperature-dependent structural changes in this region, the crystal structure was compared with the representative MD structures at 300, 320, and 340 K. In the crystal structure of PETaseSM14, the N-terminal residues Ala26 and Gln27 were oriented toward the solvent, whereas the Asn28–Asp35 residues were positioned near the α3 helix, α2–α3 loop, and α3–α4 loop. Asn28, Arg32, Gly33, and Asp35 formed a total of nine hydrogen bonds with the neighboring residues Arg78, Lys253, Asp257, Asn258, and Arg261 (Figure 6B and Table S2). In the MD structure at 300 K, the backbone conformation of the N-terminal region was similar to that observed in the crystal structure, where Asn28, Arg32, Gly33, Pro34, and Asp35 formed seven hydrogen-bond interactions with Arg78, Asp257, Asn258, and Arg261 (Figure 6B and Table S2). Although a larger number of N-terminal residues participated in the interactions with neighboring residues, the total number of hydrogen bonds was reduced compared with the crystal structure (Table S2). In the MD structures at 320 and 340 K, the position and conformation of the N-terminal region differed considerably from those in the crystal structure. At 320 K, four N-terminal residues (Pro29–Arg32) adopted a 3_10_-helical conformation, and His30, Gly33, and Asp35 formed four hydrogen-bond interactions with Lys253 and Arg261 (Figure 6B and Table S2). In addition, the N-terminus shifted toward the protein core and interacted with the core domain at 340 K, whereas the residues Asn28–Arg32 became exposed to the solvent. Furthermore, Ala26, Arg32, Gly33, and Asp35 formed seven hydrogen-bond interactions with Asp76, Asp257, and Arg261 (Figure 6B and Table S2).

## 3. Discussion

The continuous use and accumulation of PET pose a serious threat to the environment and human health [31,32]. The use of PETase enables environmentally friendly and sustainable depolymerization of PET to address plastic pollution while also facilitating the production of value-added products [7,14,33]. The enzymatic properties of PETaseSM14 under high-temperature and high-salt conditions suggest that it has potential value for future PET degradation applications [22]. In this study, MD simulations were performed to investigate the substrate recognition mechanism and molecular flexibility of PETaseSM14 to contribute to its potential protein engineering.

The MD simulations revealed that the core α/β-hydrolase fold of PETaseSM14 remained highly similar to that observed in the crystal structure across all temperatures. In contrast, local structural fluctuations and conformational changes in the active-site region exhibited temperature-dependent behavior. At 300 and 320 K, PETaseSM14 revealed conformations in which Tyr88 showed a positional shift that opened the substrate-binding cleft and exposed the catalytic Ser156 to the solvent. This rearrangement converted the closed state of the substrate-binding cleft into an open state, thereby potentially allowing access to the catalytic Ser156 residue. Notably, in the representative MD structure at 300 K, the shape of the substrate-binding cleft created by the Tyr88 position shift was highly similar to that of IsPETase, which supports the functional relevance of this conformation. These MD simulation results indicate that near the optimal temperature for enzymatic activity, PETaseSM14 dynamically opens its substrate-binding cleft via temperature-induced Tyr88 position shifts and conformational changes. Meanwhile, the HEMT docking study showed that the representative MD structure of PETaseSM14 at 300 K formed a more reliable substrate-binding pocket compared with the 320 K model, which is near the optimal temperature for enzymatic activity, indicating that structural features do not necessarily correspond directly to enzymatic activity. MD simulations evaluate structural dynamics under idealized thermodynamic conditions, and therefore, the simulation temperature does not necessarily coincide exactly with the experimentally determined optimal activity temperature [34,35]. Accordingly, rather than directly correlating the MD structural results obtained at the same temperature with enzymatic activity, it is more appropriate to interpret the findings in terms of temperature-dependent structural changes in the active site.

Temperature is a critical factor in enzymatic activity, as it can affect the protein structure’s conformation and flexibility [25,26,27]. Cryogenic crystal structures of many enzymes often exhibit biologically irrelevant molecular flexibility when compared with the structures determined at ambient or physiological temperatures [36,37]. For example, the thumb domain in the closed conformation of GH11 xylanase, which is involved in substrate recognition, exhibited a rigid conformation in cryogenic structures, whereas at room temperature, the electron density map corresponding to the thumb domain was disordered, thus indicating that molecular flexibility differed depending on the temperature [38]. In the hen egg-white lysozyme, loop regions involved in substrate recognition exhibit increased flexibility and a widened substrate-binding cleft at room temperature compared with their cryogenic structures [39]. Moreover, in xylanase TloXynII, the substrate-recognition residue Arg122 displays temperature-dependent conformational changes that influence the width of the substrate-binding cleft [40]. In addition, numerous MD studies performed over a wide temperature range have demonstrated that increasing the temperature induces conformational rearrangements and fluctuations of the proteins that provide mechanistic insight into temperature-dependent protein function [41,42,43,44,45]. Similarly, the MD results suggest that the temperature-dependent structural changes in PETaseSM14 may be associated with changes in its structural dynamics. These findings suggest that, beyond PETase, MD simulations performed under optimal temperature conditions can identify the active or substrate-recognition conformations that are not apparent in the static experimental structures. Thus, such simulations provide valuable insight into the biologically relevant structural dynamics and catalytic mechanisms across a range of enzymes.

At 340 K, which exceeds the experimentally determined Tm, the active-site architecture of PETaseSM14 changed significantly. In particular, the His183 residue, which is involved in the proton shuttle in the Ser–His–Asp catalytic triad, exhibited a conformational rearrangement that disrupted the charge-relay system. This indicates that the active site of PETaseSM14 becomes inactive before the α/β fold is disrupted, a behavior similar to that observed in other enzymes [46,47]. These findings provide a mechanistic explanation as to why enzymatic activity drops sharply near the Tm, even when the secondary structure is still partially preserved in previous circular dichroism (CD) analyses [22]. Meanwhile, CD analysis indicated that at 340 K most of the protein is unfolded [22], but the MD simulations retained the α/β fold, which suggests that the MD structure represents a pre-unfolding state. This discrepancy reflects an inherent limitation of in silico simulations, but the MD simulation results at 340 K provide insight into a possible functional inactivation state before complete protein denaturation.

The N-terminal region (Ala26–Asp35) of PETaseSM14 is located on the opposite side of the catalytic site within the α/β core and also exhibits significant temperature-dependent conformational changes and increased fluctuations. At 320 and 340 K, this region adopted distinct positions and interaction patterns with the neighboring residues, accompanied by increased flexibility. In many enzymes, catalytic activity is influenced by protein flexibility and the stability of distal regions [48,49,50]. For example, in Kemp eliminase, distal mutations that alter the structural dynamics significantly enhance catalytic efficiency by modulating the conformational fluctuations [51]. In acyltransferase LovD, remote mutations alter the backbone dynamics and shift the population of catalytically competent states, thereby affecting its activity [52]. Moreover, intrinsic protein flexibility has been recognized as a key contributor to enzymatic catalysis, thus indicating that dynamic motions beyond the active site geometry play a crucial role in their function [53]. PETase variants in other systems have been engineered through modifications of the flexible loop regions to improve their activity and thermostability [28,29,30]. Accordingly, MD simulation results suggest that engineering the N-terminal region flexibility of PETaseSM14 may represent a promising strategy for generating more efficient enzymes. In the MD structures at all temperatures, the carboxylate group of Asp35 within the N-terminal region was stabilized with the guanidinium group of Arg261 via hydrogen-bond interactions. These observations suggest that improving the stability of residues preceding Asp35 may be pivotal for maintaining the N-terminal structural rigidity. These findings may guide future strategies for engineering improved PETases. 

In a previous study, MD simulations of PETaseSM14 were performed under high salt conditions (900 mM NaCl), which exhibited a widened substrate-binding site. In this study, the MD simulations of PETaseSM14 performed without a high salt environment showed that temperature can induce rearrangement that sufficiently widens the substrate-binding pocket to allow substrate access. These MD simulation studies indicate that both salt and temperature conditions can influence the PETaseSM14 substrate-binding pocket. Future MD simulations of PETaseSM14 at functionally relevant ionic strengths and temperatures would provide further insight into the PETaseSM14 mechanism. In addition, the MD simulations in this study were analyzed based on a single trajectory due to computational limitations. To more rigorously establish statistical convergence, multiple independent replicas and longer simulations will be required in future studies.

## 4. Materials and Methods

### 4.1. Molecular Dynamics Simulations

The crystal structure of PETaseSM14 (PDB code: 9HYD) [22] was retrieved from the Protein Data Bank [54] and used as the initial model for MD simulations. All-atom MD simulations were performed using GROMACS (version 2024.4) [55] with the AMBER99SB-ILDN force field [56]. The system was solvated in a triclinic box filled with TIP3P [57] water molecules. A minimum distance of 1.0 nm was maintained between the protein surface and the box boundary. Counterions were then added to neutralize the system charge. Energy minimization was performed using the steepest descent algorithm until the maximum force was below 1000 kJ mol^−1^ nm^−1^. The system was then equilibrated in two steps, where NVT equilibration for 100 ps was performed at the target temperature using a V-rescale thermostat, followed by NPT equilibration for 100 ps using a Parrinello–Rahman barostat to maintain a pressure of 1 bar. Production MD simulations were subsequently performed under periodic boundary conditions at 300, 320, and 340 K for 200 ns. The time step was set to 2 fs, and all bonds that involved hydrogen atoms were constrained using the LINCS algorithm. Long-range electrostatic interactions were treated with the PME method with a real-space cutoff of 1.0 nm, and the van der Waals interactions were truncated at 1 nm. 

### 4.2. Trajectory Analysis

Trajectory analyses were performed using the built-in GROMACS tools and in-house scripts. The overall structural stability was evaluated by calculating the root-mean-square deviation (RMSD) of Cα atoms relative to the crystal structure. The radius of gyration (Rg) and the solvent-accessible surface area (SASA) were calculated to quantify the global compactness and surface exposure, respectively. Root-mean-square fluctuation (RMSF) was computed to evaluate the residue-wise flexibility. Secondary structure changes during the simulations were analyzed using the DSSP algorithm [58]. Representative structures of PETaseSM14 at each temperature were extracted via RMSD-based clustering of the production trajectories using a cutoff of 2.0 Å, and the most dominant cluster was then selected for structural comparison.

### 4.3. Bioinformatics

Amino acid sequence alignment was performed using Clustal Omega [59], and the structure-based sequence alignment was performed using ESPript 3.0 [60]. Calculation of the substrate binding cleft and structure-based blind docking were performed using CB-DOCK2 [61]. For structure-based blind docking, CB-Dock2 automatically identifies potential binding cavities on the protein surface and generates docking grids centered on the detected cavities. Docking calculations were performed using the AutoDock Vina scoring function implemented in CB-Dock2, which evaluates the predicted binding affinities of ligand poses. The structural figures were generated using PyMOL (http://pymol.org).

## 5. Conclusions

This study provides a temperature-dependent structural analysis of PETaseSM14 based on MD simulations. The results reveal that the closed conformation of the substrate-binding cleft opens near the optimal temperature through the positional and conformational changes in Tyr88, which leads to solvent exposure of the catalytic Ser156 residue. At elevated temperatures, disruption of the charge-relay system within the catalytic triad explains the loss of enzymatic activity before complete protein unfolding. Analysis of the flexible N-terminal region suggests that it may be a key target for improving the thermal stability thereof without compromising the flexibility required for substrate binding. Taken together, these results provide mechanistic insight into the temperature-dependent behavior of PETaseSM14 and establish a structural basis for the rational engineering of PETase variants optimized for industrial PET biodegradation.

## Data Availability

The data that support the findings of this study are available from the corresponding author upon reasonable request.

## Supplementary Materials

The following supporting information can be downloaded at: https://www.mdpi.com/article/10.3390/ijms27062825/s1.
