# Supplementary material for: Temperature-Dependent Active-Site Rearrangements of PETaseSM14: Insights from Molecular Dynamics Simulations
Source: Int J Mol Sci. 2026 Mar 20;27(6):2825. doi: 10.3390/ijms27062825 (PMC13026930; doi:10.3390/ijms27062825)
Supplement: Supplementary file 1 [file ijms-27-02825-s001.zip › ijms-4177480-supplementary.pdf]

Supplementary Data

Temperature-dependent active-site rearrangements of PETaseSM14: Insights from molecular dynamics simulations

Ki Hyun Nam

College of General Education, Kookmin University, Seoul 02707, Republic of Korea

* Corresponding: structure@kookmin.ac.kr

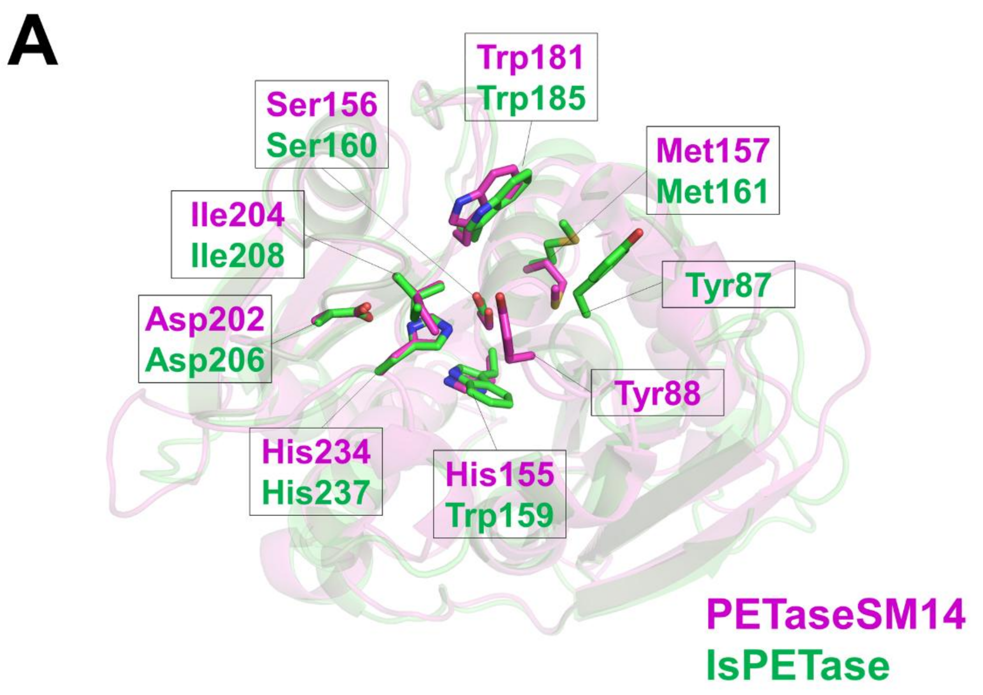

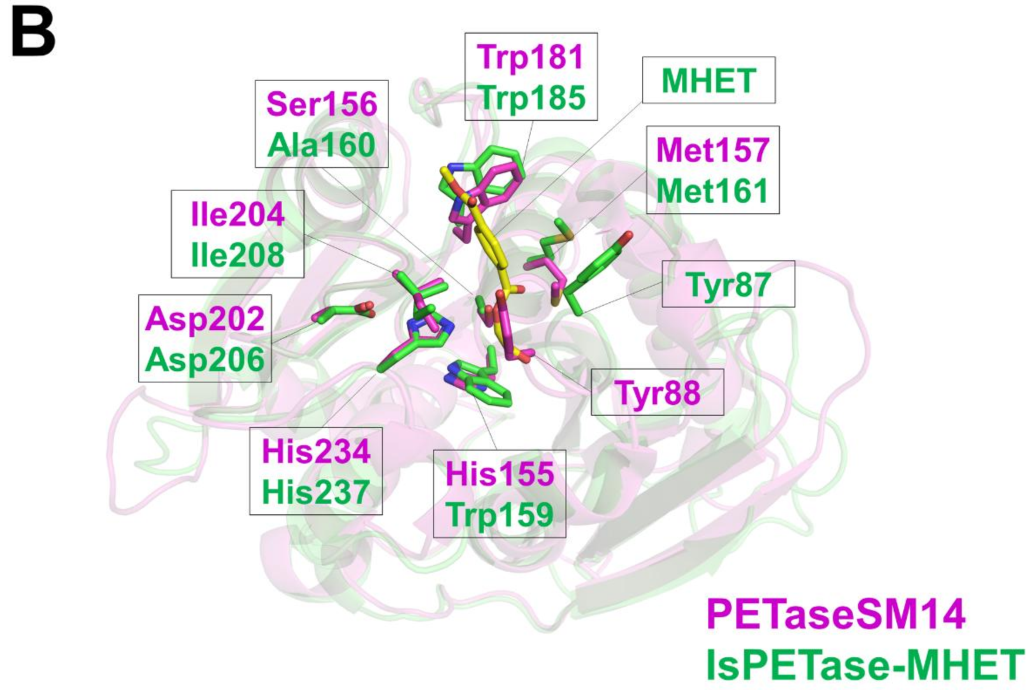

Supplementary Figure S1. Superposition of PETaseSM14 (PDB code: 9HYD) with (A) native IsPETase (5XJH) and (B) MHET-bound IsPETase (5XH3).

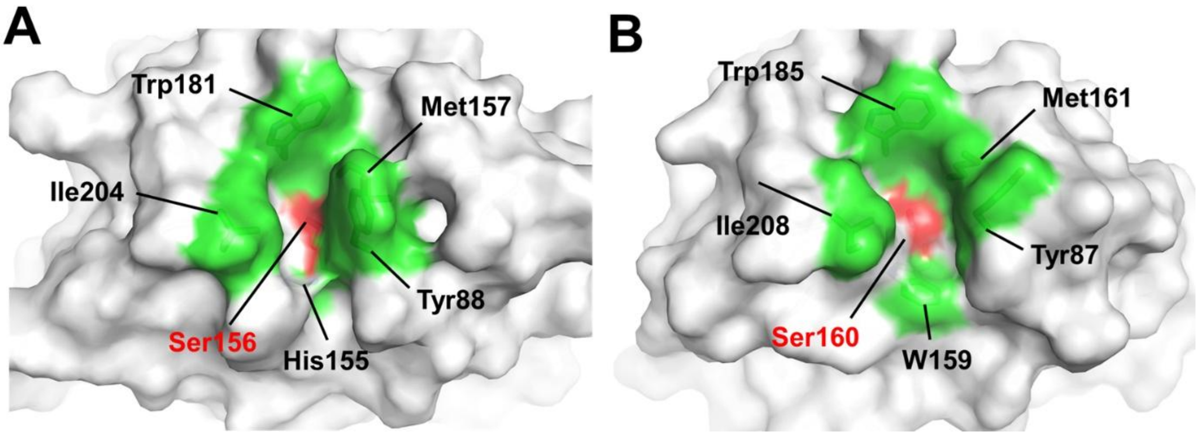

Supplementary Figure S2. Structural comparison of the substrate-binding cleft between (A) representative MD structure of PETaseSM14 at 300 K and (B) native IsPETase (PDB ID: 5XJH). Catalytic and substrate-binding cleft residues are colored red and green, respectively.

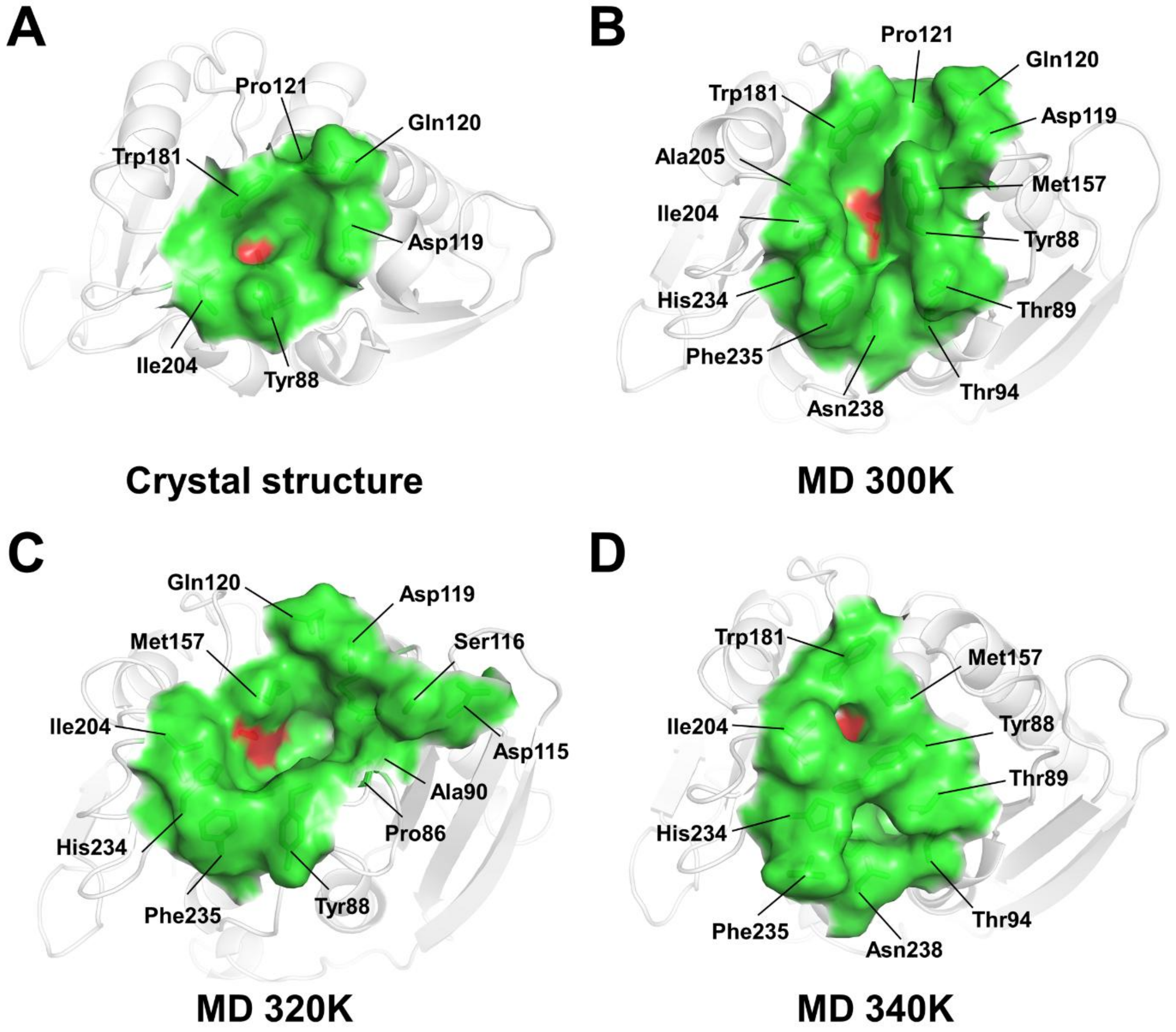

Supplementary Figure S3. Calculated substrate-binding cavity volumes of the (A) crystal structure of PETaseSM14 and representative MD structures of PETaseSM14 at (B) 300 K, (C) 320 K, and (D) 340 K. Catalytic and substrate-binding cleft residues are colored red and green, respectively.

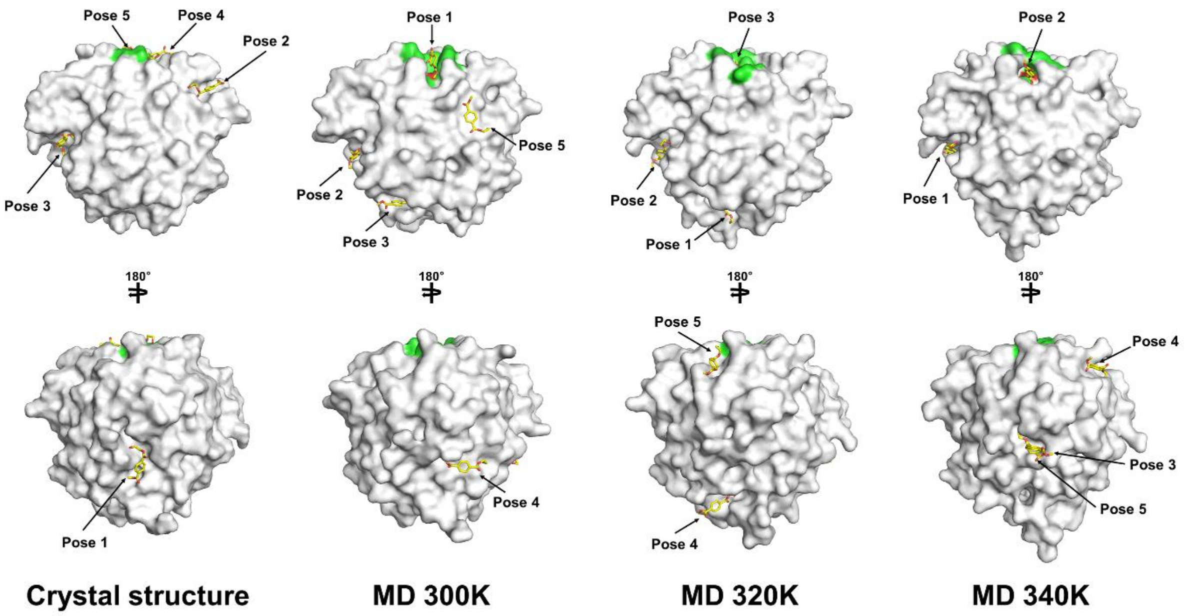

Supplementary Figure S4. Structure-based blind docking of HEMT molecule to the crystal structure and representative MD structures at 300, 320, and 340 K.

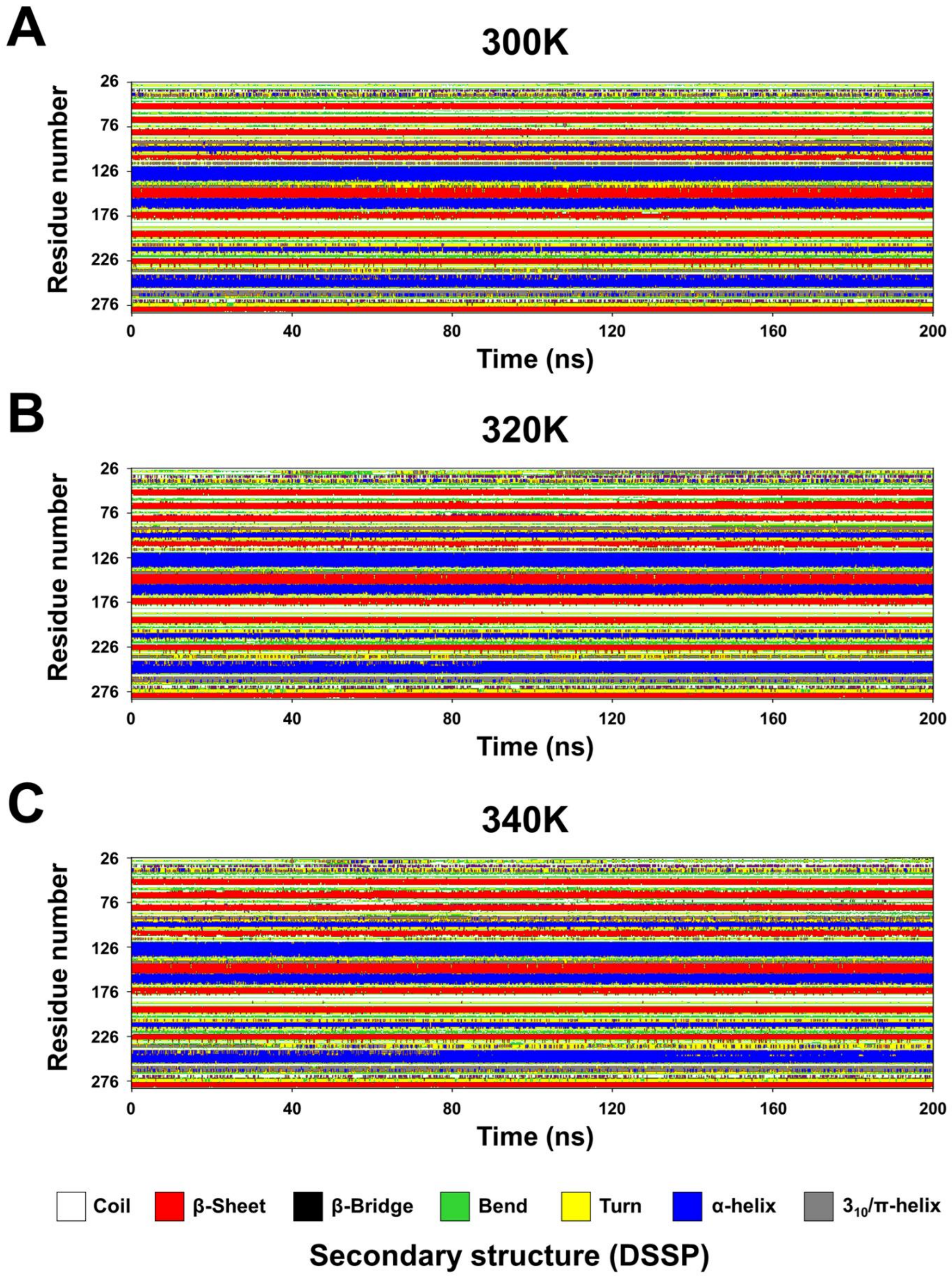

Supplementary Figure S5. DSSP secondary structure analysis as a function of simulation time for the trajectories of PETaseSM14 at (A) 300 K, (B) 320 K, and (C) 340 K.

Table S1. Summary of the structure-based blind docking.

	Crystal structure		MD 300 K		MD 320 K		MD 340 K	
Pose	Vina score	Cavity volume (Å^3)	Vina score	Cavity volume (Å^3)	Vina score	Cavity volume (Å^3)	Vina score	Cavity volume (Å^3)
1	-4.8	85	-5.5	85	-5.9	440	-5.0	393
2	-4.7	100	-5.4	324	-5.4	307	-4.8	149
3	-4.5	177	-4.5	71	-5.0	109	-4.6	123
4	-4.5	165	-4.4	264	-4.8	379	-4.6	83
5	-4.2	96	-4.4	51	-4.5	121	-4.5	167

Table S2. Intramolecular interactions of the N-terminal region in the crystal and MD structures of PETaseSM14

**Crystal structure**					
N-terminal residue (atom)	Distance (Å)	Interacting residue (atom)	N-terminal residue (atom)	Distance (Å)	Interacting residue (atom)
Asn28 (ND)	2.84	Asp257 (O)	Arg32 (O)	3.00	Arg261 (NH2)
Asn28 (ND)	3.04	Asn258 (O)	Gly33 (O)	3.03	Arg261 (NH1)
Arg32 (NE)	2.97	Arg78 (O)	Gly33 (O)	3.48	Arg261 (NH2)
Arg32 (NH2)	2.80	Arg78 (O)	Asp35 (OD2)	2.92	Arg261 (NH1)
Arg32 (O)	2.95	Lys253 (NZ)			
**MD structure at 300K**					
N-terminal residue (atom)	Distance (Å)	Interacting residue (atom)	N-terminal residue (atom)	Distance (Å)	Interacting residue (atom)
Asn28 (ND)	2.93	Asp257 (O)	Gly33 (O)	3.79	Arg261 (NH1)
Asn28 (ND)	3.23	Asn258 (O)	Pro34 (O)	2.94	Arg261 (NH1)
Arg32 (NH1)	3.15	Arg78 (O)	Asp35 (OD2)	2.89	Arg261 (NH1)
Arg32 (O)	2.88	Arg261 (NH2)			
**MD structure at 320K**					
N-terminal residue (atom)	Distance (Å)	Interacting residue (atom)	N-terminal residue (atom)	Distance (Å)	Interacting residue (atom)
His30 (O)	2.81	Lys253 (NZ)	Gly33 (O)	2.91	Arg261(NH2)
Gly33 (O)	2.79	Arg261 (NH1)	Asp35 (OD2)	3.04	Arg261(NH1)
**MD structure at 340K**					
N-terminal residue (atom)	Distance (Å)	Interacting residue (atom)	N-terminal residue (atom)	Distance (Å)	Interacting residue (atom)
Ala26 (N)	2.65	Asp76 (OD1)	Arg32 (O)	2.73	Arg261 (NH2)
Ala26 (N)	2.90	Asp257 (O)	Gly33 (O)	3.12	Arg261 (NH1)
Ala26 (N)	3.20	Asp257 (OD1)	Asp35 (OD2)	2.66	Arg261 (NH1)
Ala26 (N)	2.85	Asp257 (OD2)			
